# Breastfeeding, specific foods in the first year of life, and asthma at 6 years of age

**DOI:** 10.1590/1806-9282.20241970

**Published:** 2025-08-08

**Authors:** Vinicius Teixeira, Raphael Limas de Aguiar, Fabiana Schuelter-Trevisol, Gilvan Carneiro de Andrade, Daisson José Trevisol, Jefferson Traebert, Eliane Traebert

**Affiliations:** 1 Universidade do Sul de Santa Catarina, School of Medicine – Palhoça (SC), Brazil.; 2 Universidade do Sul de Santa Catarina, Post-Graduation Programs in Health Sciences – Palhoça (SC), Brazil.

**Keywords:** Asthma, Breastfeeding, Child nutrition, Children

## Abstract

**OBJECTIVE:**

The objective of this study was to investigate the association between breastfeeding, the introduction of specific foods in the first year of life, and asthma symptoms at 6 years of age.

**METHODS:**

A longitudinal study was conducted involving 956 children in southern Brazil. Asthma symptoms were assessed through the International Study of Asthma and Allergies in Childhood questionnaire. Information regarding breastfeeding and the introduction of complementary foods during the first year of life was obtained through interviews with mothers conducted at home. A multivariate analysis was performed using Poisson regression, and relative risks were estimated to assess the magnitude of the associations.

**RESULTS:**

Among the 956 children included in the study, 18.7% exhibited asthma symptoms. Male children had a 3% higher incidence [RR=1.03 (95%CI 1.01–1.06); p=0.023]. Children with a family history of asthma showed a 9% higher incidence [RR=1.09 (1.06–1.13); p<0.001] compared to those without such a history. Nonbreastfed children had an 8% higher incidence [RR=1.08 (95%CI 1.02–1.15); p=0.015]. Moreover, children who were introduced to wheat flour during the first year of life had a 4% higher incidence [RR=1.04 (95%CI 1.01–1.06); p=0.020] of asthma symptoms.

**CONCLUSION:**

Significant and independent associations were observed between asthma symptoms at 6 years of age and male sex, family history of asthma, breastfeeding, and the introduction of wheat flour during the first year of life.

## INTRODUCTION

Asthma, as defined by the 2024 Global Initiative for Asthma (GINA) guidelines, is a chronic respiratory condition characterized by inflammation of the airways, leading to recurrent episodes of wheezing, shortness of breath, cough, and chest tightness. These episodes are often triggered by factors such as allergens, environmental pollutants, physical exertion, and other stimuli. The condition results in airway obstruction, which is generally reversible but can become persistent if not properly managed. Asthma is commonly associated with a history of hypersensitivity and airway inflammation, making it variable in terms of symptom severity and frequency^1^.

Given the complexity of confirming asthma diagnoses in children, epidemiological studies often use questionnaires to identify asthma symptoms^2^. To enhance the value of epidemiological studies on asthma and allergic diseases, the International Study of Asthma and Allergies in Childhood (ISAAC)^3^ was developed, providing a standardized method to facilitate international collaboration.

The latest report from the GINA estimates that approximately 300 million people worldwide live with asthma. Additionally, the report warns that the prevalence of asthma has been increasing globally, particularly in urban areas, due to factors such as air pollution, climate change, and lifestyle changes^1^.

Several factors have been proposed to explain this increase, including environmental, economic, psychosocial, and particularly nutritional aspects^4,5^. Asthma is thus associated with a complex set of etiological processes. Some studies^6,7^ suggest that dietary patterns may contribute to the increased prevalence of respiratory complications and asthma in children.

Recently, the role of diet, particularly during the first 2 years of life, has been implicated in asthma development, with breastfeeding identified as a significant protective factor^8^. Exclusive breastfeeding for 4 months reduces the risk of asthma between four and 6 years of age^9^. Conversely, the early introduction of cow's milk after discontinuing exclusive breastfeeding is associated with asthma diagnosis in this age group^9^. Lima et al.^10^ showed evidence regarding the role of nutrient-rich foods and how their gradual introduction may help modulate the immune response, with a particular focus on avoiding early exposure to specific food allergens, which could be crucial in preventing sensitizations and the development of asthma in childhood. Additionally, the consumption of fruits and vegetables and adherence to the Mediterranean diet are protective factors against asthma^11^.

On the other hand, the increasing prevalence of ultraprocessed foods in family diets may contribute to higher asthma incidence in children due to the poor nutritional quality of these foods^12^.

However, studies exploring the relationship between factors influencing childhood asthma remain limited^13^. Thus, investigating this topic is crucial, as improper dietary habits during early life can lead to chronic inflammatory diseases such as asthma later. Understanding the association between the introduction of various food types in the first 2 years of life and asthma onset may help prevent the disease and improve children's quality of life. This study aimed to examine the possible association between the introduction of different foods during the first year of life and asthma symptoms at 6 years of age.

## METHODS

A longitudinal epidemiological study was conducted using data from the *Coorte Brasil Sul* cohort^14^. This cohort study was carried out in Palhoça, SC, a municipality in the Brazilian Southern state of Santa Catarina with a population of approximately 227,000 inhabitants.

The study population comprised children born in 2009 who were followed up until 2015, when they were 6 years old. The minimum sample size calculation for this study used the following parameters: a population of 1,756 children, an anticipated prevalence of asthma symptoms (p=50%), a 95% confidence level, and a 4% relative error, resulting in a minimum sample size of 448 children. The dataset contained information on 956 children, and all were included in this study.

The *Coorte Brasil Sul* study^14^ collected data through interviews using the ISAAC questionnaire^2^ and structured instruments for sociodemographic information. Interviews were conducted with the mothers in their homes or, in their absence, with the child's primary caregiver. Data collection was carried out by the *Coorte Brasil Sul* ^14^ research team, supported by trained community health agents from Palhoça, SC. Duplicate data collection was performed on 5% of the sample population, selected randomly, to monitor the reproducibility of the information.

The dependent variable in this study was the mother's report of asthma symptoms at 6 years of age, based on the ISAAC questionnaire^3^ question: "Has your child had wheezing in the chest in the last 12 months?" (yes or no). Independent variables included: child's sex (male or female); skin color (white or non-white); maternal education at birth (up to eight years of schooling or more than eight years); family history of asthma (yes or no); breastfeeding (yes or no); presence of smokers in the household (yes or no); and the consumption of specific foods in the first year of life: eggs, wheat products (e.g., pasta, bread, and cake), soy, corn, seafood, and fish (yes or no).

The dataset was prepared using Excel software and analyzed with SPSS version 18.0 (SPSS Inc., Chicago, IL., USA). Variables were described using proportions and 95% confidence intervals. Bivariate analysis was conducted using the chi-square test, with a significance level of p<0.05. The multivariate analysis included variables with p<0.20 in the bivariate analysis and used Poisson regression. Variables with p<0.05 were considered significant and independent. Relative risk (RR) and 95% confidence intervals were used as measures of association. The research project was approved by the Ethics Committee of the Universidade do Sul de Santa Catarina, Brazil, under the approval number 38240114.0.0000.5369.

## RESULTS

A total of 956 6-year-old children participated in the study, of whom 491 (51.4%) were male and 798 (83.3%) were white. Additional sociodemographic variables are shown in Table 1.

**Table 1:** Results of the association study between asthma symptom reports at 6 years of age and sociodemographic variables, breastfeeding, family history of asthma, and smoking within the household (Palhoça, SC, Brazil).

Variables		Asthma symptoms			p-value
		Yes	No	Total	
		n (%)	n (%)	n (%)	
Sex (n=956)					
	Male	103 (21.0)	388 (79.0)	491 (51.4)	0.066
	Female	76 (16.3)	389 (83.7)	465 (48.6)	
Skin color (n=956)					
	Non-white	40 (25.0)	120 (75.0)	160 (16.7)	0.025
	White	88 (17.5)	417 (82.5)	505 (83.3)	
Maternal education at birth (years) (n=901)					
	≤8 years	139 (17.5)	657 (82.5)	796 (46.3)	0.094
	>8 years	88 (21.1)	329 (78.9)	417 (53.7)	
Breastfeeding (n=956)					
	Yes	81 (16.7)	403 (83.3)	484 (91.1)	<0.001
	No	150 (17.2)	721 (82.8)	871 (8.9)	
Family history of asthma (n=946)					
	Yes	28 (32.9)	57 (67.1)	85 (45.7)	<0.001
	No	121 (28.0)	311 (72.0)	432 (54.3)	
Smoking within household (n=956)					
	Yes	54 (25.7)	136 (74.3)	183 (19.2)	0.007
	No	132 (17.1)	641 (82.9)	773 (80.8)	

The reported prevalence of asthma symptoms in the past 12 months was 18.7% (95%CI 16.2–21.1), with associations observed for skin color (p=0.025), breastfeeding (p<0.001), family history of asthma (p<0.001), and the presence of smokers in the household (p=0.007) (Table 1). Table 2 presents data on the introduction of specific foods in the first year of life, with wheat products showing a significant association with asthma symptoms (p=0.006).

**Table 2:** Results of the association study between the introduction of specific foods in the first year of life and asthma symptoms at 6 years of age (Palhoça, SC, Brazil).

Variables		Asthma symptoms			p-value
		Yes	No	Total	
		n (%)	n (%)	n (%)	
Cow's milk and dairy products (n=949)					
	Yes	162 (18.7)	704 (81.3)	866 (91.2)	0.899
	No	16 (19.3)	67 (80.7)	83 (8.8)	
Wheat flour (n=956)					
	Yes	133 (21.3)	493 (78.7)	626 (65.5)	0.006
	No	46 (13.9)	284 (86.1)	330 (34.5)	
Soy and derivatives (n=953)					
	Yes	152 (19.2)	638 (80.8)	790 (82.9)	0.198
	No	25 (15.3)	138 (84.7)	163 (17.1)	
Corn and derivatives (n=956)					
	Yes	41 (23.2)	136 (76.8)	177 (18.5)	0.090
	No	138 (17.7)	641 (82.3)	779 (81.5)	
Chicken egg (n=947)					
	Yes	95 (19.8)	384 (80.2)	479 (50.6)	0.318
	No	81 (17.3)	387 (82.7)	468 (49.4)	
Fish (n=956)					
	Yes	70 (18.8)	303 (81.2)	373 (39.0)	0.959
	No	163 (18.9)	702 (81.1)	865 (90.5)	
Seafood (n=956)					
	Yes	109 (18.7)	474 (81.3)	583 (61.0)	0.777
	No	16 (17.6)	75 (82.4)	91 (9.5)	

The multivariate analysis results are detailed in Table 3. Boys had a 3% higher incidence of asthma symptoms than girls, which was statistically significant and independent [RR=1.03 (95%CI 1.01–1.06); p=0.023]. Similarly, children with a family history of asthma had a 9% higher incidence of symptoms compared to those without a family history [RR=1.09 (95%CI 1.06–1.13); p<0.001]. Nonbreastfed children had an 8% higher incidence [RR=1.08 (95%CI 1.02–1.15); p=0.015], and children introduced to wheat products before 1 year of age had a 4% higher incidence [RR=1.04 (95%CI 1.01–1.06); p=0.020].

**Table 3:** Results of the multivariate analysis between asthma symptom reports at 6 years of age and the studied independent variables (Palhoça, SC, Brazil).

Variables		RR_c_ (95%CI)	p-value	RR_a_ (95%CI)	p-value
Sex					
	Female	1.00	0.066	1.00	0.023
	Male	1.03 (0.99; 1.05)		1.03 (1.01; 1.06)	
Skin color					
	White	1.00	0.025	1.00	0.118
	Non-white	1.04 (1.01; 1.09)		1.03 (0.99–1.08)	
Maternal education at birth (years)					
	>8 years	1.00	0.094	1.00	0.270
	≤8 years	1.02 (0.99; 1.05)		1.02 (0.99–1.05)	
Family history of asthma					
	No	1.00	<0.001	1.00	<0.001
	Yes	1.10 (1.07; 1.13)		1.09 (1.06–1.13)	
Breastfeeding					
	Yes	1.00	<0.001	1.00	0.015
	No	1.09 (1.03; 1.16)		1.08 (1.02–1.15)	
Smoking within household					
	No	1.00	0.007	1.00	0.428
	Yes	1.05 (1.01; 1.09)		1.02 (0.98–1.06)	
Introduction of wheat flour in the first year of life					
	No	1.00	0.006	1.00	0.020
	Yes	1.04 (1.01; 1.07)		1.04 (1.01–1.06)	
Introduction of soy and derivatives in the first year of life					
	No	1.00	0.198	1.00	0.449
	Yes	1.03 (0.98; 1.07)		1.01 (0.97–1.07)	
Introduction of corn and derivatives in the first year of life					
	No	1.00	0.090	1.00	0.971
	Yes	1.03 (0.99; 1.07)		1.01 (0.96–1.05)	

RR_c_: crude relative risk; RR_a_: adjusted relative risk; 95%CI: 95% confidence interval. Omnibus test p=0.757.

## DISCUSSION

This study found an 18.7% prevalence of asthma symptoms among 6-year-olds, using the ISAAC questionnaire^3^. This rate is lower than that reported in a study^15^ conducted in São Paulo, SP, Brazil, using a similar methodology, which observed a prevalence of 31.2%. Similar studies conducted in 20 other Brazilian cities among the same age group reported prevalences of 24.4% in Manaus, AM, 29.9% in Natal, RN, and 20.6% in Itajaí, SC^15^.

While this study's methods do not allow for definitive explanations of the prevalence differences, sociodemographic characteristics and the time elapsed between studies may account for these contrasts. Environmental factors, particularly air pollution, have been associated with higher asthma prevalence in urban areas^16^.

Asthma is a complex, multifactorial disease characterized by chronic airway inflammation, with genetic and environmental determinants of its allergic phenotype^1^. Factors such as sex, family history of asthma, breastfeeding, and the introduction of dietary allergens in the first year of life are associated with asthma^17,18^.

In the Holt et al. study^17^, boys had a 3% higher incidence of asthma symptoms than girls, consistent with evidence that being male is a risk factor in childhood, although this trend reverses in adolescence^17^. This sex-related difference may be explained by narrower airways in boys during childhood, though this remains a controversial topic^17^.

Family genetics also play a critical role in asthma, with parental asthma being a strong predictor of asthma in children^19^. Studies indicate that children are three times more likely to develop asthma if one parent has the disease and six times more likely if both parents are affected^18^. This study supports this finding, as it observed that children with a family history of asthma had a 9% higher incidence of asthma symptoms compared to those without a family history of the disease.

Breastfeeding emerged as a protective factor against asthma. Breast milk provides immunological components, such as IgA and IgG, which contribute to passive immunization, as well as anti-inflammatory agents, immunomodulators, and essential nutrients absent in nonmaternal milk^19^. Introducing nonmaternal milk before four months of age increases asthma and atopy risks later in childhood^20^. Furthermore, delaying the introduction of nonbreast milk until at least four months of age may protect against asthma and atopy later in childhood^21^. Accordingly, this study aligns with previous research, highlighting breastfeeding as a protective factor against asthma, showing that nonbreastfed children had an 8% higher incidence of asthma.

This study also found a significant association between the introduction of wheat products before one year of age and asthma symptoms at 6 years. Wheat is one of the most common triggers of allergic reactions in children due to proteins such as albumins, globulins, and gliadins, which activate the immune system^22,23^. A study^24^ found a 64% incidence of asthma in children sensitized to gliadin. Additionally, another study^25^ on childhood allergies demonstrated that children sensitized to six common food allergens (egg, milk, soy, peanut, wheat, and fish) had higher rates of asthma-related hospitalizations. This aligns with the present study, which showed that children introduced to wheat flour in their diet before the age of one had a 4% higher incidence of asthma symptoms. However, in this study, no statistically significant and independent associations were observed with the introduction of cow's milk and its derivatives, soy and its derivatives, corn and its derivatives, chicken eggs, fish, or seafood during the first year of life.

While these findings are significant, some limitations should be noted, such as potential recall bias in caregiver reports and insufficient depth in exploring certain variables. The use of the ISAAC [3] allows for symptom reporting but not an asthma diagnosis. Moreover, some variables require more in-depth investigation to better clarify their actual influence on the final outcome, such as whether breastfeeding was exclusively maternal or supplemented. Longitudinal studies are needed to further investigate the role of early dietary factors in asthma development, including foods for which there is some evidence, but which were not confirmed in this study.

In conclusion, this study identified higher asthma symptom incidence among boys, children with a family history of asthma, nonbreastfed children, and those introduced to wheat products before 1 year of age.

## Data Availability

The datasets generated and/or analyzed during the current study are available from the corresponding author upon reasonable request.

## References

[1] Global Initiative for Asthma (GINA) (2024). Global strategy for asthma management and prevention.

[2] Pearce N, Aït-Khaled N, Beasley R, Mallol J, Keil U, Mitchell E (2007). Worldwide trends in the prevalence of asthma symptoms: phase III of the International Study of Asthma and Allergies in Childhood (ISAAC). Thorax.

[3] Asher MI, Keil U, Anderson HR, Beasley R, Crane J, Martinez F (1995). International Study of Asthma and Allergies in Childhood (ISAAC): rationale and methods. Eur Respir J.

[4] Andrade LS, Araújo ACTB, Cauduro TM, Watanabe LA, Castro APBM, Jacob CMA (2013). Obesidade e asma: associação ou epifenômeno?. Rev Paul Pediatr.

[5] Bezerra CMFMC, Trabulsi RK, Furtado CD, Matos IM, Batista LVO, Vieira JC (2024). Manejo da Asma na pediatria:epidemiologia, diagnóstico e tratamento. Braz J Health Rev.

[6] D’Innocenzo S, Matos SM, Prado MS, Santos CA, Assis AM, Cruz AA (2014). [Dietary pattern, asthma, and atopic and non-atopic wheezing in children and adolescents: SCAALA study, Salvador, Bahia State, Brazil]. Cad Saude Publica.

[7] Emmanouil E, Manios Y, Grammatikaki E, Kondaki K, Oikonomou E, Papadopoulos N (2010). Association of nutrient intake and wheeze or asthma in a Greek pre-school population. Pediatr Allergy Immunol.

[8] Andrade JFL, Traebert E, Garcia LP, Traebert J (2022). Occurrence of infectious diseases during the first two years and symptoms of asthma in six-year-old children. Res Soc Develop.

[9] Schneider AP, Stein RT, Fritscher CC (2007). The role of breastfeeding, diet and nutritional status in the development of asthma and atopy. J Bras Pneumol.

[10] Franco JM, Oliveira LCL, Castro APBM, Pomiecinski F, Reali ACR, Yang AC (2022). Introdução dos alimentos no primeiro ano de vida e prevenção da alergia alimentar: quais as evidências?. Arq Asma Alerg Imunol.

[11] Takkinsatian P, Mairiang D, Sangkanjanavanich S, Chiewchalermsri C, Tripipitsiriwat A, Sompornrattanaphan M (2022). Dietary factors associated with asthma development: a narrative review and summary of current guidelines and recommendations. J Asthma Allergy.

[12] Serra HCOA, Rudakoff LCS, Muniz AKOA, Magalhães EIDS, Bragança MLBM, Silva AAMD (2023). Association between the Consumption of Ultra-Processed Foods and Asthma in Adults from Ribeirão Preto, São Paulo, Brazil. Nutrients.

[13] Lodge CJ, Tan DJ, Lau MX, Dai X, Tham R, Lowe AJ (2015). Breastfeeding and asthma and allergies: a systematic review and meta-analysis. Acta Paediatr.

[14] Traebert J, Lunardelli SE, Martins LGT, Santos KD, Nunes RD, Lunardelli AN (2018). Methodological description and preliminary results of a cohort study on the influence of the first 1,000 days of life on the children's future health. An Acad Bras Cienc.

[15] Casagrande RR, Pastorino AC, Souza RG, Leone C, Solé D, Jacob CM (2008). [Asthma prevalence and risk factors in schoolchildren of the city of São Paulo, Brazil]. Rev Saude Publica.

[16] Watts J (2006). Doctors blame air pollution for China's asthma increases. Lancet.

[17] Holt PG, Upham JW, Sly PD (2005). Contemporaneous maturation of immunologic and respiratory functions during early childhood: implications for development of asthma prevention strategies. J Allergy Clin Immunol.

[18] Xue M, Dehaas E, Chaudhary N, O’Byrne P, Satia I, Kurmi OP (2021). Breastfeeding and risk of childhood asthma: a systematic review and meta-analysis. ERJ Open Res.

[19] Pérez-Losada M, Castro-Nallar E, Bendall ML, Freishtat RJ, Crandall KA (2015). Dual transcriptomic profiling of host and microbiota during health and disease in pediatric asthma. PLoS One.

[20] Danielewicz H (2022). Breastfeeding and allergy effect modified by genetic, environmental, dietary, and immunological factors. Nutrients.

[21] Oddy WH, Holt PG, Sly PD, Read AW, Landau LI, Stanley FJ (1999). Association between breast feeding and asthma in 6 year old children: findings of a prospective birth cohort study. BMJ.

[22] Longo G, Berti I, Burks AW, Krauss B, Barbi E (2013). IgE-mediated food allergy in children. Lancet.

[23] Tatham AS, Shewry PR (2008). Allergens to wheat and related cereals. Clin Exp Allergy.

[24] Kotaniemi-Syrjänen A, Palosuo K, Jartti T, Kuitunen M, Pelkonen AS, Mäkelä MJ (2010). The prognosis of wheat hypersensitivity in children. Pediatr Allergy Immunol.

[25] Mansouri M, Pourpak Z, Mozafari H, Abdollah Gorji F, Shokouhi Shoormasti R (2012). Follow-up of the wheat allergy in children; consequences and outgrowing the allergy. Iran J Allergy Asthma Immunol.

